# Transactions of the American Medical Association

**Published:** 1872-03

**Authors:** 


*Transactions of the American Medical Association, held at San Francisco, Cal., May,* 1871.

Volume XXII of the transactions of the American Medical Association comes to us filled with much interesting matter. The meeting at San Francisco was somewhat disturbed by the "Woman Question," which is spoken of to some length in the able address of the President, Dr. Alfred Stille. The Committee on Medical Education, presents an exhaustive and able report, and suggest some excellent reforms in the manner of educating young men for the medical profession. Many of the papers presented to the Association show commendable care and thought; and all are valuable and interesting.

